# CARE FOR OLDER ADULTS: WHAT SKILLS DO HEALTH CARE EMPLOYERS VALUE IN COMMUNITY COLLEGE STUDENTS?

**DOI:** 10.1093/geroni/igad104.0772

**Published:** 2023-12-21

**Authors:** Runcie Chidebe, Phyllis Cummins, Donnette Narine, Takashi Yamashita

**Affiliations:** Miami University, Oxford, Ohio, United States; Miami University, Oxford, Ohio, United States; University of Maryland Baltimore, Baltimore, Maryland, United States; University of Maryland, Baltimore County, Baltimore, Maryland, United States

## Abstract

Community colleges (CCs) play an important role in educating healthcare workers engaged in caring for aging populations with greater healthcare and long-term care needs. Healthcare employers and nursing home leaders are obligated to ensure that care provided by their employees meets quality standards, is patient-centered and free from psychological and physical harm. To increase the employability of CC students and understand the skills valued by employers in improving care for older adults, we explored the role literacy, numeracy, and problem-solving skills play in CC student success in the workplace. Using a qualitative descriptive design, we conducted semi-structured interviews with CC administrators (n=9), faculty (n=6), and employers (n=10) who hire healthcare workers (certified nursing assistants, licensed practical nurses (LPNs), and registered nurses (RNs)) educated at CCs. Our findings show that communication skills are highly valued, especially the ability to communicate on the level of the nursing home resident or hospital patient, along with their family members. Numeracy skills, such as measuring medication dosages, critical thinking skills, and problem-solving skills, including conflict resolution, emotional intelligence, and compassionate care are valued by employers. Employers reported that LPNs and RNs have limited leadership and supervisory skills and there is a need to use innovative mechanisms (e.g., case studies, online courses) to improve those skills. Serving on CC advisory councils, clinical rotations and employers’ input into program development were reported as beneficial strategies. Findings from this project will inform both CC administrators and employers regarding skills that are valued in caring for older adults.

